# Crystal structure of 2-bromo-4,6-di­nitroaniline

**DOI:** 10.1107/S2056989015017946

**Published:** 2015-10-03

**Authors:** Gihaeng Kang, Tae Ho Kim, Eui-Jae Lee, Chang Ho Kang

**Affiliations:** a Department of Chemistry and Research Institute of Natural Sciences, Gyeongsang, National University, Jinju 52828, Republic of Korea; b Research Center at Kyung-In Synthetic Corporation (KISCO), Yangcheon-ro 75-69, Gangseo-gu, Seoul 07517, Republic of Korea; c Division of Applied Life Science and PMBBRC, Gyeongsang, National University, Jinju 52828, Republic of Korea

**Keywords:** crystal structure, aniline derivative, hydrogen bonding, C—Br⋯π inter­actions

## Abstract

In the title compound, C_6_H_4_BrN_3_O_4_, the dihedral angles between the nitro groups and the aniline ring are 2.04 (3) and 1.18 (4)°, respectively. In the crystal, N—H⋯O and C—H⋯O hydrogen bonds and weak side-on C—Br⋯π inter­actions [3.5024 (12) Å] link adjacent mol­ecules, forming a three-dimensional network. A close O⋯Br contact [3.259 (2) Å] may also add additional stability.

## Related literature   

For information on the title compound, see: Yadav & Sharma (2010 ▸). For a related crystal structure, see: Glidewell *et al.* (2002 ▸).
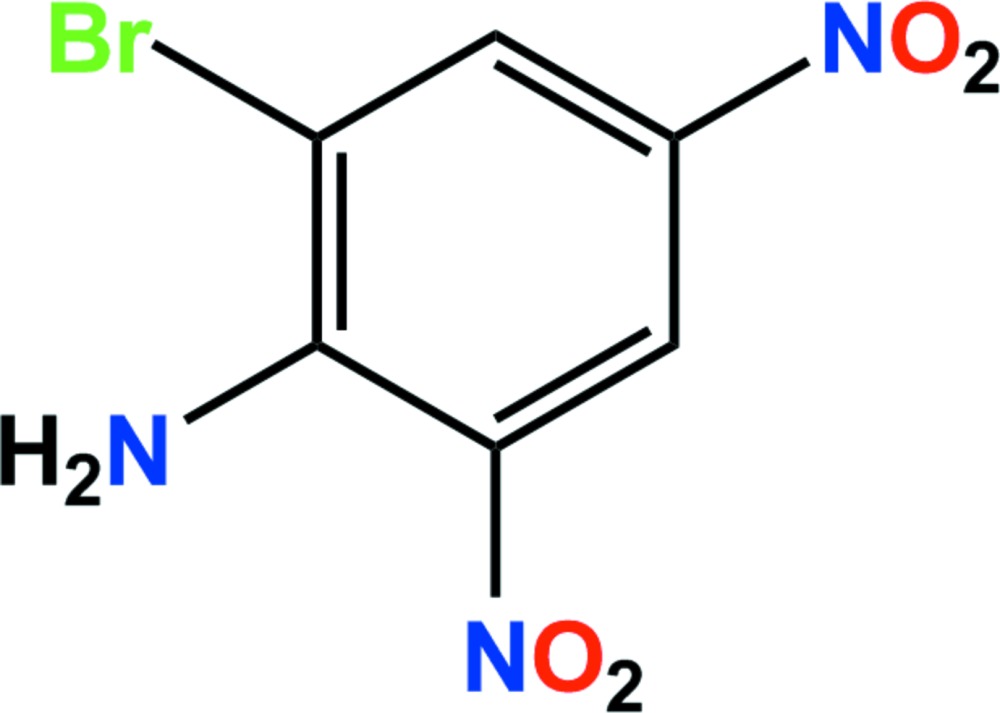


## Experimental   

### Crystal data  

C_6_H_4_BrN_3_O_4_
*M* *_r_* = 262.03
Monoclinic 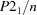

*a* = 6.6955 (2) Å
*b* = 7.7720 (2) Å
*c* = 16.0608 (4) Å
β = 95.4182 (14)°
*V* = 832.03 (4) Å^3^
*Z* = 4
Mo *K*α radiation
μ = 4.93 mm^−1^
*T* = 173 K
0.20 × 0.15 × 0.08 mm

### Data collection  

Bruker APEXII CCD diffractometer
Absorption correction: multi-scan (*SADABS*; Bruker, 2013 ▸) *T*_min_ = 0.534, *T*_max_ = 0.746
12322 measured reflections
1892 independent reflections
1648 reflections with *I* > 2σ(*I*)
*R*_int_ = 0.030

### Refinement  

*R*[*F* ^2^ > 2σ(*F* ^2^)] = 0.033
*wR*(*F* ^2^) = 0.083
*S* = 1.06
1892 reflections
127 parameters
H-atom parameters constrained
Δρ_max_ = 0.85 e Å^−3^
Δρ_min_ = −0.51 e Å^−3^

### 

Data collection: *APEX2* (Bruker, 2013 ▸); cell refinement: *SAINT* (Bruker, 2013 ▸); data reduction: *SAINT*; program(s) used to solve structure: *SHELXS97* (Sheldrick, 2008 ▸); program(s) used to refine structure: *SHELXL2013* (Sheldrick, 2015 ▸); molecular graphics: *DIAMOND* (Brandenburg, 2010 ▸); software used to prepare material for publication: *SHELXTL* (Sheldrick, 2008 ▸).

## Supplementary Material

Crystal structure: contains datablock(s) global, I. DOI: 10.1107/S2056989015017946/sj5477sup1.cif

Structure factors: contains datablock(s) I. DOI: 10.1107/S2056989015017946/sj5477Isup2.hkl

Supporting information file. DOI: 10.1107/S2056989015017946/sj5477Isup3.cml

**Fig. 1.**
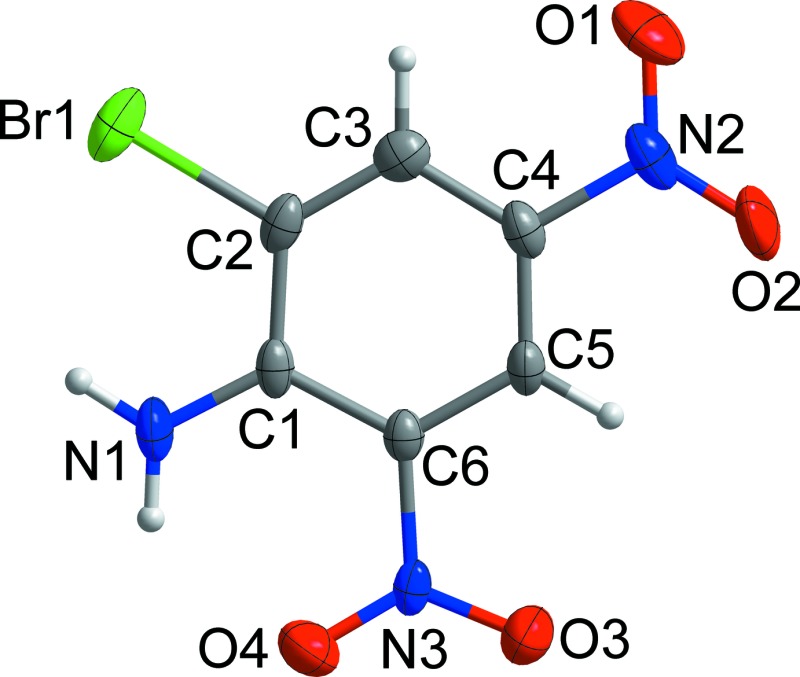
The asymmetric unit of the title compound with the atom-numbering scheme. Displacement ellipsoids are drawn at the 50% probability level. H atoms are shown as small spheres of arbitrary radius.

**Fig. 2.**
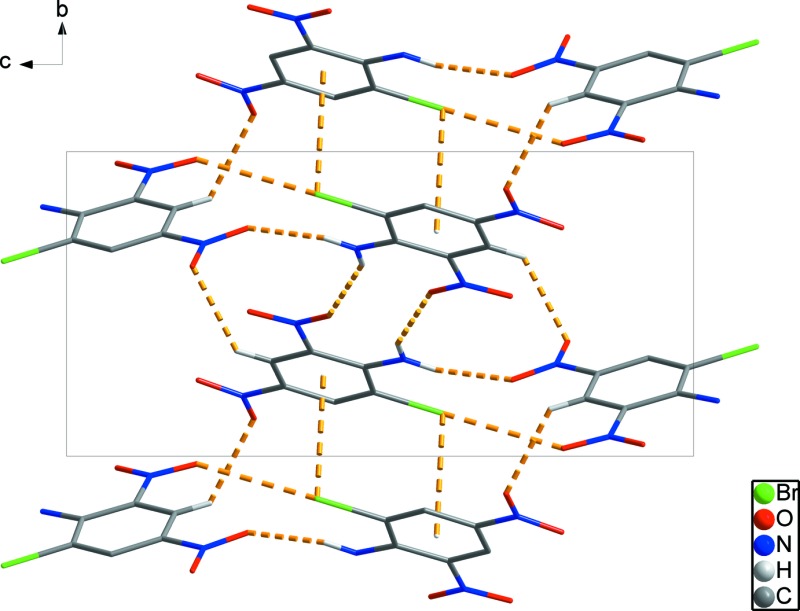
Crystal packing viewed along the a axis. The intermolecular interactions are shown as dashed lines.

. DOI: 10.1107/S2056989015017946/sj5477fig1.tif
The asymmetric unit of the title compound with the atom-numbering scheme. Displacement ellipsoids are drawn at the 50% probability level. H atoms are shown as small spheres of arbitrary radius.

a . DOI: 10.1107/S2056989015017946/sj5477fig2.tif
Crystal packing viewed along the *a* axis. The inter­molecular inter­actions are shown as dashed lines.

CCDC reference: 1427403

Additional supporting information:  crystallographic information; 3D view; checkCIF report

## Figures and Tables

**Table 1:** Hydrogen-bond geometry (, )

*D*H*A*	*D*H	H*A*	*D* *A*	*D*H*A*
N1H1*A*O2^i^	0.88	2.16	2.893(3)	141
N1H1*B*O4^ii^	0.88	2.36	3.139(4)	148
C5H5O1^iii^	0.95	2.55	3.209(4)	127

Symmetry codes: (i) 
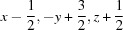
; (ii) 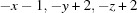; (iii) 
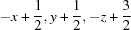
.

## supplementary crystallographic information

### S1. Comment

The title compound, C_6_H_4_BrN_3_O_4_, is an aniline derivative with additional bromine and nitro substituents. Aniline is the simplest of the primary aromatic amines an organic base used, as are its derivatives, to make dyes, drugs, explosives, plastics and chemicals for the rubber industry (Yadav & Sharma, 2010). Its crystal structure is reported herein. In this compound (Fig. 1), the dihedral angles between the nitro groups and the aniline ring are 2.04 (3) and 1.18 (4)°, respectively. All bond lengths and bond angles are normal and comparable to those observed in the crystal structure of a similar compound (Glidewell *et al.*, 2002).

The crystal structure (Fig. 2) is stabilized by N—H···O and C—H···O hydrogen bonds (Table 1), as well as an intermolecular side-on C2—Br1···*Cg*1^iv^ interaction [Br1···Cg = 3.5024 (12) Å, C2—Br1···Cg = 96.90 (9) °] (*Cg*1 is the centroid of the C1–C6 ring) [symmetry code: (iv), -*x*, -*y* + 1, -*z* + 2]. A close O3···Br1^iv^ contact, 3.259 (2) Å may also contribute, iv = -1/2+x,1.5-y, -1/2+z. These contacts result in a three-dimensional network.

### S2. Experimental

The title compound was supplied by the Kyung In Synthetic Corporation. Slow evaporation of a solution in CH_2_Cl_2_ gave single crystals suitable for X-ray analysis.

### S3. Refinement

All H-atoms were positioned geometrically and refined using a riding model with d(N—H) = 0.88 Å, *U*_iso_ = 1.2*U*_eq_(C) for amine group, d(C—H) = 0.95 Å, *U*_iso_ = 1.2*U*_eq_(C) for aromatic C—H.

### Crystal data

**Table:**

C_6_H_4_BrN_3_O_4_	*F*(000) = 512
*M**_r_* = 262.03	*D*_x_ = 2.092 Mg m^−^^3^
Monoclinic, *P*2_1_/*n*	Mo *K*α radiation, λ = 0.71073 Å
*a* = 6.6955 (2) Å	Cell parameters from 6099 reflections
*b* = 7.7720 (2) Å	θ = 2.6–27.1°
*c* = 16.0608 (4) Å	µ = 4.93 mm^−^^1^
β = 95.4182 (14)°	*T* = 173 K
*V* = 832.03 (4) Å^3^	Block, yellow
*Z* = 4	0.20 × 0.15 × 0.08 mm

### Data collection

**Table:**

Bruker APEXII CCD diffractometer	1648 reflections with *I* > 2σ(*I*)
φ and ω scans	*R*_int_ = 0.030
Absorption correction: multi-scan (*SADABS*; Bruker, 2013)	θ_max_ = 27.5°, θ_min_ = 2.6°
*T*_min_ = 0.534, *T*_max_ = 0.746	*h* = −8→8
12322 measured reflections	*k* = −10→10
1892 independent reflections	*l* = −20→20

### Refinement

**Table:**

Refinement on *F*^2^	0 restraints
Least-squares matrix: full	Hydrogen site location: inferred from neighbouring sites
*R*[*F*^2^ > 2σ(*F*^2^)] = 0.033	H-atom parameters constrained
*wR*(*F*^2^) = 0.083	*w* = 1/[σ^2^(*F*_o_^2^) + (0.0334*P*)^2^ + 1.6027*P*] where *P* = (*F*_o_^2^ + 2*F*_c_^2^)/3
*S* = 1.06	(Δ/σ)_max_ = 0.002
1892 reflections	Δρ_max_ = 0.85 e Å^−^^3^
127 parameters	Δρ_min_ = −0.51 e Å^−^^3^

### Special details

**Table:**

Geometry. All e.s.d.'s (except the e.s.d. in the dihedral angle between two l.s. planes) are estimated using the full covariance matrix. The cell e.s.d.'s are taken into account individually in the estimation of e.s.d.'s in distances, angles and torsion angles; correlations between e.s.d.'s in cell parameters are only used when they are defined by crystal symmetry. An approximate (isotropic) treatment of cell e.s.d.'s is used for estimating e.s.d.'s involving l.s. planes.

### Fractional atomic coordinates and isotropic or equivalent isotropic displacement parameters (Å^2^)

**Table:**

	*x*	*y*	*z*	*U*_iso_*/*U*_eq_	
Br1	0.19258 (6)	0.64018 (4)	1.10024 (2)	0.04116 (14)	
O1	0.4575 (4)	0.6238 (3)	0.79915 (19)	0.0484 (7)	
O2	0.2420 (4)	0.7493 (4)	0.71047 (15)	0.0544 (8)	
O3	−0.3569 (3)	0.9745 (3)	0.79238 (13)	0.0379 (5)	
O4	−0.4360 (3)	0.9614 (3)	0.91874 (15)	0.0397 (6)	
N1	−0.1949 (4)	0.8183 (4)	1.03776 (15)	0.0327 (6)	
H1A	−0.1531	0.7837	1.0886	0.039*	
H1B	−0.3120	0.8696	1.0282	0.039*	
N2	0.2978 (4)	0.6977 (4)	0.78113 (17)	0.0348 (6)	
N3	−0.3276 (3)	0.9358 (3)	0.86609 (13)	0.0212 (5)	
C1	−0.0806 (4)	0.7930 (4)	0.97511 (16)	0.0230 (6)	
C2	0.1093 (4)	0.7101 (4)	0.98987 (17)	0.0256 (6)	
C3	0.2302 (4)	0.6777 (4)	0.92827 (19)	0.0277 (6)	
H3	0.3551	0.6208	0.9404	0.033*	
C4	0.1671 (4)	0.7299 (4)	0.84695 (18)	0.0263 (6)	
C5	−0.0114 (4)	0.8115 (4)	0.82743 (17)	0.0233 (6)	
H5	−0.0513	0.8456	0.7715	0.028*	
C6	−0.1333 (4)	0.8435 (3)	0.89051 (16)	0.0214 (5)	

### Atomic displacement parameters (Å^2^)

**Table:**

	*U*^11^	*U*^22^	*U*^33^	*U*^12^	*U*^13^	*U*^23^
Br1	0.0571 (2)	0.0375 (2)	0.02593 (18)	−0.01145 (16)	−0.01191 (14)	0.00718 (13)
O1	0.0422 (14)	0.0411 (14)	0.0655 (18)	0.0096 (11)	0.0239 (13)	−0.0063 (12)
O2	0.0471 (15)	0.094 (2)	0.0241 (12)	−0.0027 (15)	0.0165 (11)	−0.0089 (13)
O3	0.0415 (13)	0.0450 (14)	0.0268 (11)	0.0051 (11)	0.0004 (10)	0.0036 (10)
O4	0.0329 (12)	0.0438 (14)	0.0437 (14)	0.0057 (10)	0.0111 (11)	0.0000 (11)
N1	0.0409 (15)	0.0416 (15)	0.0168 (11)	−0.0026 (12)	0.0096 (11)	−0.0002 (11)
N2	0.0351 (15)	0.0381 (15)	0.0340 (15)	−0.0082 (12)	0.0174 (12)	−0.0124 (12)
N3	0.0269 (12)	0.0220 (11)	0.0147 (10)	−0.0105 (9)	0.0011 (9)	0.0003 (9)
C1	0.0323 (15)	0.0214 (13)	0.0156 (12)	−0.0095 (11)	0.0051 (11)	−0.0032 (10)
C2	0.0339 (15)	0.0246 (14)	0.0175 (13)	−0.0095 (12)	−0.0025 (11)	0.0019 (11)
C3	0.0274 (14)	0.0242 (14)	0.0308 (15)	−0.0045 (11)	−0.0010 (12)	−0.0003 (12)
C4	0.0290 (15)	0.0289 (15)	0.0224 (13)	−0.0054 (12)	0.0100 (12)	−0.0058 (11)
C5	0.0276 (14)	0.0274 (14)	0.0153 (12)	−0.0069 (11)	0.0046 (11)	−0.0009 (10)
C6	0.0257 (13)	0.0218 (13)	0.0170 (12)	−0.0045 (11)	0.0031 (10)	−0.0019 (10)

### Geometric parameters (Å, º)

**Table:**

Br1—C2	1.887 (3)	N3—C6	1.505 (4)
O1—N2	1.224 (4)	C1—C2	1.425 (4)
O2—N2	1.228 (4)	C1—C6	1.426 (4)
O3—N3	1.219 (3)	C2—C3	1.360 (4)
O4—N3	1.182 (3)	C3—C4	1.395 (4)
N1—C1	1.335 (4)	C3—H3	0.9500
N1—H1A	0.8800	C4—C5	1.363 (4)
N1—H1B	0.8800	C5—C6	1.382 (4)
N2—C4	1.456 (4)	C5—H5	0.9500
			
C1—N1—H1A	120.0	C1—C2—Br1	117.8 (2)
C1—N1—H1B	120.0	C2—C3—C4	118.6 (3)
H1A—N1—H1B	120.0	C2—C3—H3	120.7
O1—N2—O2	123.7 (3)	C4—C3—H3	120.7
O1—N2—C4	118.7 (3)	C5—C4—C3	122.2 (3)
O2—N2—C4	117.6 (3)	C5—C4—N2	119.1 (3)
O4—N3—O3	126.8 (3)	C3—C4—N2	118.7 (3)
O4—N3—C6	117.9 (2)	C4—C5—C6	118.7 (3)
O3—N3—C6	115.3 (2)	C4—C5—H5	120.6
N1—C1—C2	120.5 (3)	C6—C5—H5	120.6
N1—C1—C6	124.7 (3)	C5—C6—C1	122.6 (3)
C2—C1—C6	114.8 (2)	C5—C6—N3	116.8 (2)
C3—C2—C1	123.1 (3)	C1—C6—N3	120.6 (2)
C3—C2—Br1	119.1 (2)		
			
N1—C1—C2—C3	178.6 (3)	C3—C4—C5—C6	0.1 (4)
C6—C1—C2—C3	−1.3 (4)	N2—C4—C5—C6	−179.2 (3)
N1—C1—C2—Br1	−0.5 (4)	C4—C5—C6—C1	−0.7 (4)
C6—C1—C2—Br1	179.52 (19)	C4—C5—C6—N3	179.3 (2)
C1—C2—C3—C4	0.9 (4)	N1—C1—C6—C5	−178.7 (3)
Br1—C2—C3—C4	180.0 (2)	C2—C1—C6—C5	1.3 (4)
C2—C3—C4—C5	−0.2 (4)	N1—C1—C6—N3	1.3 (4)
C2—C3—C4—N2	179.1 (3)	C2—C1—C6—N3	−178.7 (2)
O1—N2—C4—C5	−179.7 (3)	O4—N3—C6—C5	178.5 (3)
O2—N2—C4—C5	1.4 (4)	O3—N3—C6—C5	−0.6 (3)
O1—N2—C4—C3	1.0 (4)	O4—N3—C6—C1	−1.5 (4)
O2—N2—C4—C3	−177.9 (3)	O3—N3—C6—C1	179.4 (3)

### Hydrogen-bond geometry (Å, º)

**Table:**

*D*—H···*A*	*D*—H	H···*A*	*D*···*A*	*D*—H···*A*
N1—H1*A*···O2^i^	0.88	2.16	2.893 (3)	141
N1—H1*B*···O4^ii^	0.88	2.36	3.139 (4)	148
C5—H5···O1^iii^	0.95	2.55	3.209 (4)	127

Symmetry codes: (i) *x*−1/2, −*y*+3/2, *z*+1/2; (ii) −*x*−1, −*y*+2, −*z*+2; (iii) −*x*+1/2, *y*+1/2, −*z*+3/2.
