# Supplementary figures and images for: Crystal structure of 2-bromo-4,6-di­nitroaniline
Source: Acta Crystallogr E Crystallogr Commun. 2015 Oct 3;71(Pt 11):o813. doi: 10.1107/S2056989015017946 (PMC4645020; doi:10.1107/S2056989015017946)

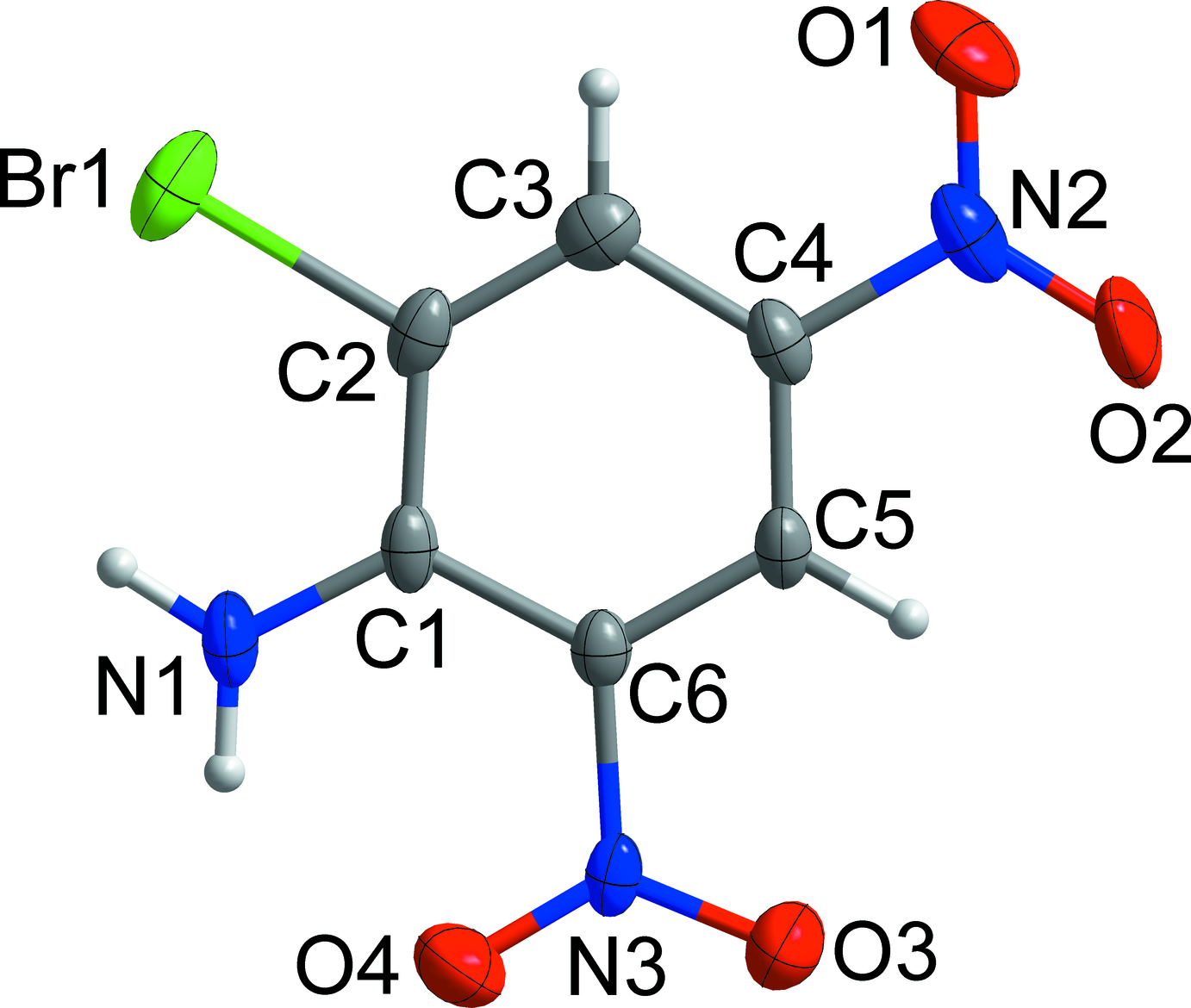

Supplement: Supplementary file 4 [file e-71-0o813-fig1.tif]

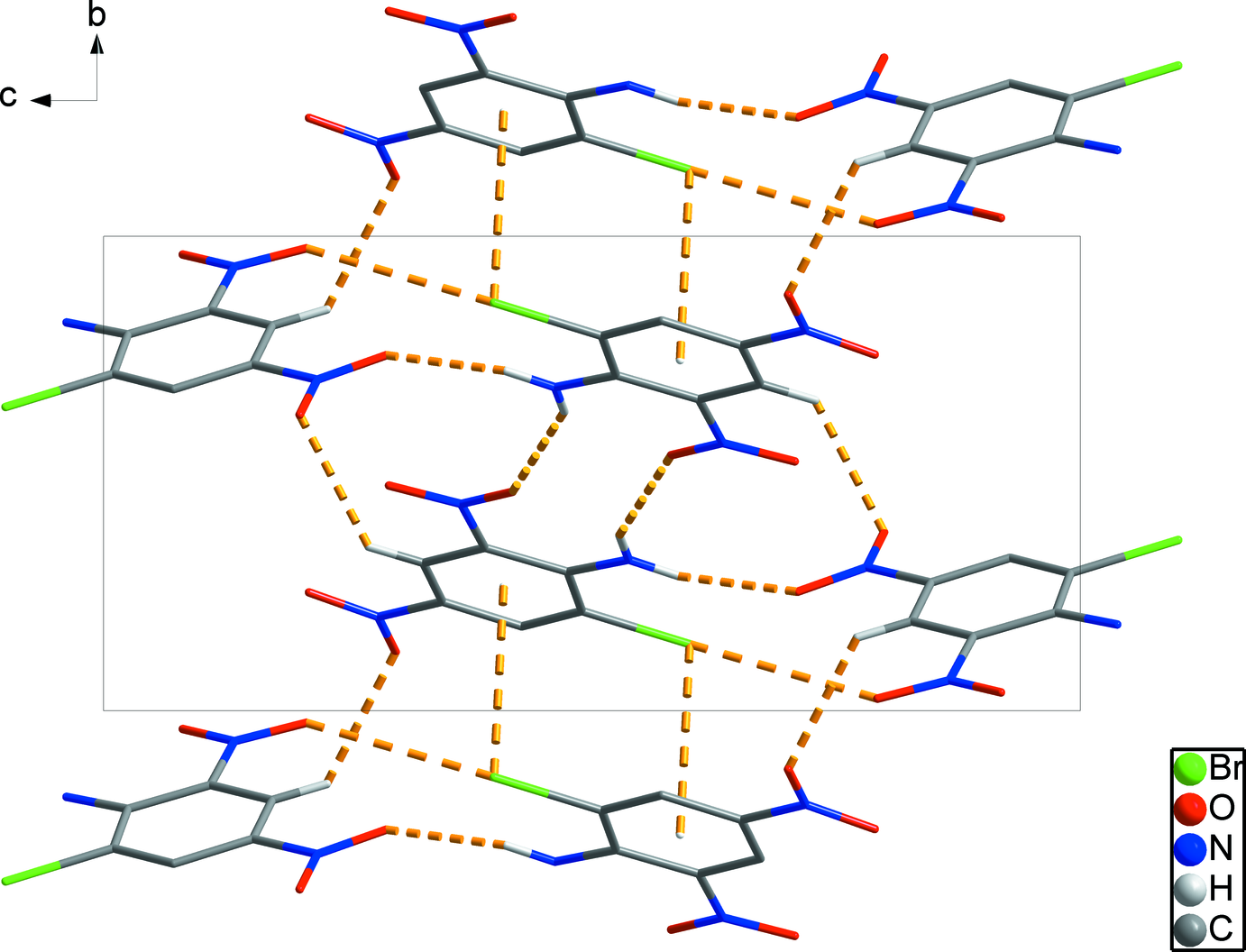

Supplement: Supplementary file 5 [file e-71-0o813-fig2.tif]
